# Effect of Culture Temperature on 2-Methylisoborneol Production and Gene Expression in Two Strains of *Pseudanabaena* sp.

**DOI:** 10.3390/cells13161386

**Published:** 2024-08-20

**Authors:** Rumi Park, Mi-Na Yu, Ji-Hyun Park, Taegu Kang, Jung-Eun Lee

**Affiliations:** 1 Han River Environment Research Center, National Institute of Environmental Research, Yangpyeong 12585, Republic of Korea; prm3270@korea.kr (R.P.); zmffjq2435@korea.kr (M.-N.Y.); jihpark24@korea.kr (J.-H.P.); taegu98@korea.kr (T.K.); 2 Division of Water Supply and Sewerage Research, National Institute of Environment Research, Incheon 22689, Republic of Korea

**Keywords:** 2-methylisoborneol, *Pseudanabaena*, gene expression, culture temperature, strain specificity

## Abstract

The presence of the odorant 2-methylisoborneol (2-MIB) in drinking water sources is undesirable. Although 2-MIB production is known to be influenced by temperature, its regulation at the gene level and its relationship with Chlorophyll-*a* (Chl-*a*) at different temperatures remain unclear. This study investigates the impact of temperature on 2-MIB production and related gene expression in *Pseudanabaena* strains PD34 and PD35 isolated from Lake Paldang, South Korea. The strains were cultured at three temperatures (15, 25, and 30 °C) to examine cell growth, 2-MIB production, and *mic* gene expression levels. 2-MIB production per cell increased with higher temperatures, whereas *mic* gene expression levels were higher at lower temperatures, indicating a complex regulatory mechanism involving post-transcriptional and enzyme kinetics factors. Additionally, the relationship between Chl-*a* and 2-MIB involved in metabolic competition was analyzed, suggesting that high temperatures appear to favor 2-MIB synthesis more than Chl-*a* synthesis. The distinct difference in the total amount of the two products and the proportion of 2-MIB between the two strains partially explains the variations in 2-MIB production. These findings highlight the significant effect of temperature on 2-MIB biosynthesis in *Pseudanabaena* and provide a valuable background for gene data-based approaches to manage issues regarding 2-MIB in aquatic environments.

## 1. Introduction

Odor compounds synthesized by microorganisms in surface water ecosystems are receiving continuous attention worldwide. Numerous studies have highlighted the issues caused by these odorants, including increased expenses for water treatment plants and consumer distrust of the safety of drinking water [1,2,3,4,5]. Among these odorants, 2-methylisoborneol (2-MIB) and geosmin are major contributors to earthy/musty odors in drinking water sources [6]. The structural properties of 2-MIB and its lower aqueous solubility result in the poor removal efficiency of 2-MIB in several oxidation processes compared with geosmin [7,8].

2-MIB is a secondary metabolite produced by various microorganisms, including actinomycetes, myxobacteria, fungi, and cyanobacteria [9,10,11,12]. 2-MIB production by cyanobacteria has been associated with temperature and eutrophication [1]. The temperature can influence cyanobacterial metabolic reactions, leading to a shift in algal communities to bloom-forming cyanobacteria [13,14] and altered cellular productivity and 2-MIB release [3,15,16].

Several *Pseudanabaena* species possess the biosynthetic genes for 2-MIB production. A close correlation has been reported between the 2-MIB concentration and *Pseudanabaena* cell density [17,18,19]. Although optimal growth temperatures vary by species, *Pseudanabaena* exhibits a broad temperature tolerance, surviving in both cultured and freshwater conditions. Wang and Li [20] and Shen et al. [21] reported 2-MIB production by *Pseudanabaena* sp. under lab-scale conditions, ranging from 10 °C to 35 °C. Remarkably, Khan et al. [22] observed an increase in the cell density of *Pseudanabaena catenata* at 4 °C. Additionally, field work by Gao et al. [23] confirmed that *Pseudanabaena* sp. was dominant during February and March at certain sampling sites. These findings suggest that the detection of 2-MIB may be directly related to the occurrence of *Pseudanabaena* sp. not only in the summer but also in the spring and autumn [24,25].

Some filamentous cyanobacteria, including *Pseudanabaena*, possess two genes *mtf* and *mic*, encoding geranyl diphosphate (GPP) methyltransferase (GPPMT) and 2-MIB synthase (MIBS), respectively, which catalyze the formation of 2-MIB via two serial reactions in the isoprenoid pathway [26,27]. GPPMT methylates GPP to form 2-methyl-GPP, which is subsequently cyclized by MIBS to 2-MIB in the presence of Mg^2+^. Some studies [11,21] have shown that *mtf* and *mic* have similar expression patterns under the same conditions. To further understand the biosynthetic activity of 2-MIB at the gene level, we targeted the *mic* gene.

Chlorophyll-*a* (Chl-*a*) is commonly used as an indicator of cyanobacterial biomass in aquatic environments. Chl-*a* is significant in the study of 2-MIB and geosmin because it shares part of the biosynthetic pathway with these compounds. GPP is an intermediate precursor of Chl-*a* and the monoterpene precursor of 2-MIB, suggesting a competitive relationship between Chl-*a* and 2-MIB [28]. Indeed, opposing trends between 2-MIB and Chl-*a* have been observed under certain environmental conditions [3,29]. Thus, to understand the relationship between 2-MIB and Chl-*a* at different temperatures, in the current study, we considered Chl-*a* as a competitor to 2-MIB rather than merely as biomass.

To date, few studies have reported on gene regulation by temperature at the RNA level for 2-MIB production. By quantifying both DNA and RNA using digital PCR (dPCR), we confirmed the 2-MIB synthesis potential and expression level of the *mic* gene in *Pseudanabaena* sp.

The production dynamics of odorants exhibit specificity within species and even among strains of odor-producing cyanobacteria [30,31]. However, strain-specific variations of odorants within the same species have been rarely studied. In this study, we cultured two strains of *Pseudanabaena* sp. that are major producers of 2-MIB; these strains were collected from different locations on different dates and cultured under laboratory conditions. We focused on the effect of temperature on the 2-MIB productivity of these two strains, examining the metabolic relationship between 2-MIB and Chl-*a* and the expression of the *mic* gene. Additionally, we explored the distinct characteristics of the two strains and the common influence of culture temperature on the biosynthesis of 2-MIB.

## 2. Materials and Methods

### 2.1. Identification of the Bacteria and Mic Gene

Two strains of *Pseudanabaena* sp., isolated from Lake Paldang, South Korea, were used as 2-MIB-producing cyanobacteria. Individual filaments of *Pseudanabaena* were collected using the pipetting method described by Belcher and Swale [32] and subsequently cultured. To identify the cultured *Pseudanabaena*, the 16S rRNA gene fragment was amplified using the 27F/1492R primers. The 16S rRNA sequences obtained from the two strains, coded as *Pseudanabaena* sp. PD34 and *Pseudanabaena* sp. PD35, were deposited in GenBank at NCBI (National Center for Biotechnology Information). A phylogenetic tree was constructed using 16S rRNA sequences of *Pseudanabaena* species closely related to these two strains retrieved from the NCBI GenBank database (Figure S1). The phylogenetic analysis indicated that PD34 and PD35 are variants of the species *Pseudanabaena foetida*. The presence of the 2-MIB synthase gene (*mic*) in both strains was determined using the MIB3324F/MIB4050R primers as described by Suurnäkki et al. [33]. Details of all primers/probes used in this study are presented in Table 1.

### 2.2. Culture Experiments

The strains were grown in 200 mL of BG11 medium [34] in a shaking incubator set at 100 rpm under 32 µmol photons/s/m^2^ (12-hour light/dark cycle). The incubation temperatures were set to 15, 25, and 30 °C, reflecting the temperature points at which *Pseudanabaena* sp. and 2-MIB were significantly observed in the Lake Paldang basin [35]. The experiments were carried out over 7 weeks (8 time points), with one culture per week used for analysis from a total of nine independent cultures, including a control culture. To verify repeatability, the cultivation was carried out in duplicate under the same conditions. Chl-*a* and cell density (cells/mL) were measured as indicators of cell growth. Chl-*a* was analyzed as described by Lee and Gil [36] using a Cary 3500 UV/Vis spectrophotometer (Agilent, Santa Clara, CA, USA) to measure absorbance at 630, 645, 663, and 750 nm.

### 2.3. DNA and RNA Extraction

Cultured cyanobacteria were collected and immediately filtered using a membrane filter (0.45 µm pore size; Merck Millipore, Burlington, MA, USA). DNA and RNA were extracted from the filtered cells using the DNeasy PowerWater Kit and RNeasy PowerWater Kit (Qiagen, Hilden, Germany), respectively, following the manufacturer’s protocols, with an additional cell lysis step at 65 °C for 10 min. All samples were extracted with 100 µL of the elution solution. RNA purity was confirmed by measuring the A260/A280 ratio using a Nanodrop spectrophotometer (Thermo Scientific, Waltham, MA, USA) immediately after extraction.

### 2.4. dPCR Setup for Mic Gene Quantification

dPCR was used for quantifying target DNA/RNA genes due to its precision and higher detection sensitivity for low-copy-number nucleic acids [37]. Eluted nucleic acids stored at −70 °C were analyzed using the QIAcuity nanoplate-based dPCR system (Qiagen) with QIAcuity Software Suite V2.5.0.

The PCR targeted the *mic* gene in the 2-MIB operon. The *mic* gene was specifically amplified using the 3909F/4028R primers [38] and a TaqMan probe (3987P) with 6-carboxyfluorescein (FAM) and black hole quencher 1 (BHQ-1) (Table 1). To normalize *mic* gene expression, the CYAN 328R probe and the CYAN 108F/CYAN 377R primers were used as *Pseudanabaena*-specific primers/probe sets, modified from Rinta-Kanto et al. [39].

**Table 1:** Primers and probes used in this study.

Primers /Probes	Target	Sequence (5′→3′)	Target Size (bp)	Ta (°C)	Reference
27F	Universal bacteria	AGAGTTTGATYMTGGCTCAG	>1200	56	Sung et al. [40]
1492R		TACGGYTACCTTGTTACGACT			
CYAN 108F	*Pseudanabaena*- specific 16S rRNA	ACGGGTGAGTAACRCGTRA	270	55	Modified from Rinta-Kanto et al. [39]
CYAN 377R		CCATTGCGGAAAATTCCCC			
CYAN 328R		FAM-CTCAGTTCCAGTGTGACTGGTC-BHQ1			
MIB3324F	Cyanobacterial MIB synthase	CATTACCGAGCGATTCAACGAGC	726	52	Suurnäkki et al. [33]
MIB4050R		CCGCAATCTGTAGCACCATGTTGA			
3909F	Cyanobacterial MIB synthase (*mic*)	CACCAGATCTTTTCTTCGATC	140	59	Lee et al. [38]
4028R		AATCTGTAGCACCATGTTGAC			
3987P		FAM-TCCTTTCGGTTGCCA-BHQ1			this study

For DNA analysis, a 40 µL reaction mixture was prepared containing 10 µL of 4× Probe Master Mix, 1.6 µL of each primer (10 µM), 0.8 µL of probe, and 4 µL of template DNA. The thermal cycling conditions included an initial denaturation step at 95 °C for 2 min, followed by 50 cycles of 95 °C for 15 s and 59 °C for 30 s (primer annealing/extension).

For RNA analysis, the QIAcuty OneStep Advanced Probe Kit (Qiagen) was used for one-step reverse transcription dPCR (RT-dPCR). The 40 µL reaction mixture included 10 µL of 4× OneStep Advanced Probe Master Mix, 0.4 µL of 100× OneStep RT Mix, 1.6 µL of each primer (10 µM), 0.8 µL of probe (10 µM), and 5 µL of RNA extract. After a 40 min reverse transcription step at 50 °C, dPCR was performed with an initial heat activation at 95 °C for 2 min, followed by 50 cycles of 95 °C for 5 s and 59 °C (or 55 °C) for 30 s. The concentration of target genes (copies/mL) in the samples was calculated using the following equation:

$$\mathrm{Concentration}\;\mathrm{of}\;\mathrm{target}\;\mathrm{gene}\;\left(\mathrm{copies}/\mathrm{mL}\right)\;=\frac{V_{R}}{V_{T}}\times \frac{\mathrm{Elution}\;V\;\left(\mu L\right)}{\mathrm{Filtration}\;V\;\left(\mathrm{mL}\right)}\times \mathrm{Instrument}\;\mathrm{value}\;\left(\mathrm{copies}/\mu L\right)$$

where V_R_ and V_T_ represent the total reaction volume and template volume in dPCR, respectively. In this equation, the filtered volume of the cultivation sample (Filtration V) and the extracted volume of DNA or RNA (Elution V) were considered.

### 2.5. GC-MS Analysis of 2-MIB

The concentration of 2-MIB was analyzed using a gas chromatography system (Varian 450-GC; Agilent, USA) equipped with mass spectrometry (Agilent 5977B; Agilent, USA). Detection of total 2-MIB followed a modified method by Hurlburt et al. [41]. Samples were treated via headspace solid-phase microextraction (HS-SPME) with a CombiPAL autosampler before injection into the GC-MS system. A 10 mL sample was stirred with 3 g of NaCl and incubated at 70 °C for 30 min, allowing volatile organic compounds, including 2-MIB, to transfer to the headspace of the vial. These compounds were adsorbed onto a 50/30 µm DVB/CAR/PDMS SPME fiber (Supelco, Bellefonte, PA, USA) and extracted by heating at 270 °C for 4 min. Separated compounds were measured qualitatively and quantitatively based on their physicochemical properties using the DB-5MS column in the GC and the MS detector. A calibration curve prepared with a spiked standard mixed solution (47525-U; Supelco, Bellefonte, PA, USA) was used to determine the concentration of 2-MIB in the samples.

### 2.6. Statistical Analysis

Statistical analysis was carried out to evaluate the effect of temperature on 2-MIB production and differences among cyanobacterial strains. Significant differences between data groups were determined using one-way analysis of variance (ANOVA) and independent *t*-tests. Statistical significance was set at *p* < 0.05. Data statistics were analyzed using SPSS Statistics v.21 software (IBM, Chicago, IL, USA).

## 3. Results and Discussion

### 3.1. Cell Growth, 2-MIB Production, and Mic Gene Abundance

**Figure 1:**
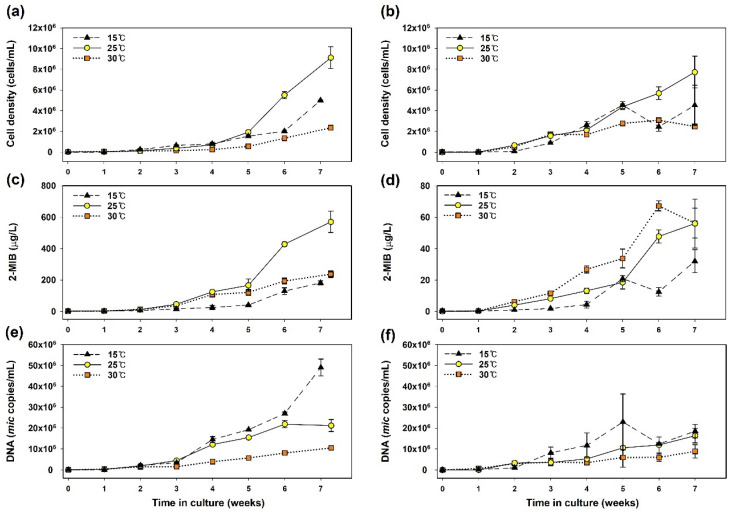
Temporal variations in components in the *Pseudanabaena* cultures: (**a**,**b**) cell density; (**c**,**d**) the concentration of 2-MIB; and (**e**,**f**) *mic* gene abundance in DNA samples. The left and right plots represent *Pseudanabaena* sp. PD34 and PD35, respectively.

The two strains of *Pseudanabaena* sp., both 2-MIB producers, exhibited different patterns of cell growth and odorant production under the three temperatures (15 °C, 25 °C, and 30 °C). For both strains, the maximum cell density was highest at 25 °C and lowest at 30 °C (Figure 1a,b). At the optimal growth temperature of 25 °C, the specific growth rate of PD34 was 0.12 d^−1^, which was higher than PD35’s rate of 0.07 d^−1^. An unusual drop in cell density was observed after 6 weeks at 15 °C; this appears to be an error during the random selection and analysis of independent cultures (Figure 1b).

The levels of each variable related to 2-MIB increased over time with cell growth. Notably, significant differences in 2-MIB production were observed between the strains. The 2-MIB concentration with PD34 was 10-fold higher than that with PD35 (evident from the y-axis scale in Figure 1c,d). The maximum 2-MIB concentrations with PD34 were 181 μg/L, 570 μg/L, and 237 μg/L at 15 °C, 25 °C, and 30 °C, respectively. For PD35, the maximum concentrations were 32 μg/L, 56 μg/L, and 67 μg/L at 15 °C, 25 °C, and 30 °C, respectively.

Interestingly, the abundance of the *mic* gene in the DNA samples did not correlate with 2-MIB concentrations at the three temperatures during the incubation period (Figure 1e,f). The 2-MIB concentration was the lowest, while the *mic* gene copy number was the highest for both strains at 15 °C compared to those at other temperatures. The *mic* gene copies did not increase proportionally with cell growth across temperatures. At 30 °C, both cell density and *mic* gene copy number were low, but the gene abundance at 15 °C exceeded that at 25 °C, which is the temperature that showed optimal growth. The average DNA retention per cell in the exponential phase was 10–11 copies/cell for PD34 and 3–7 copies/cell for PD35, with the highest abundance at 15 °C. Although it may vary depending on the growth or metabolic state, the effect of temperature on DNA content has not been verified [42,43]. Our results show that the *mic* DNA content can vary slightly with temperature and with strain.

Watson et al. [9] reviewed odorant yields per unit quantity of cyanobacteria across various studies, showing a wide range of geosmin and 2-MIB production yields among genera or species in lab cultivation. Production yields can vary with physiological states such as growth phase and environmental stress [44,45]. During the decay period, cell death leads to the release of cell-bound 2-MIB into the extracellular space [3,46]. Furthermore, the odorant productivity of cyanobacteria may be overestimated in the early stages of cultivation due to insufficient population. In this study, 2-MIB production by *Pseudanabaena* was measured during the exponential phase (from week 2 onwards), which will be discussed in the following section. The exponential phase was determined by referencing the linear portion of the semi-log graph of cell growth.

### 3.2. The Effect of Temperature on 2-MIB Production Yield

**Figure 2:**
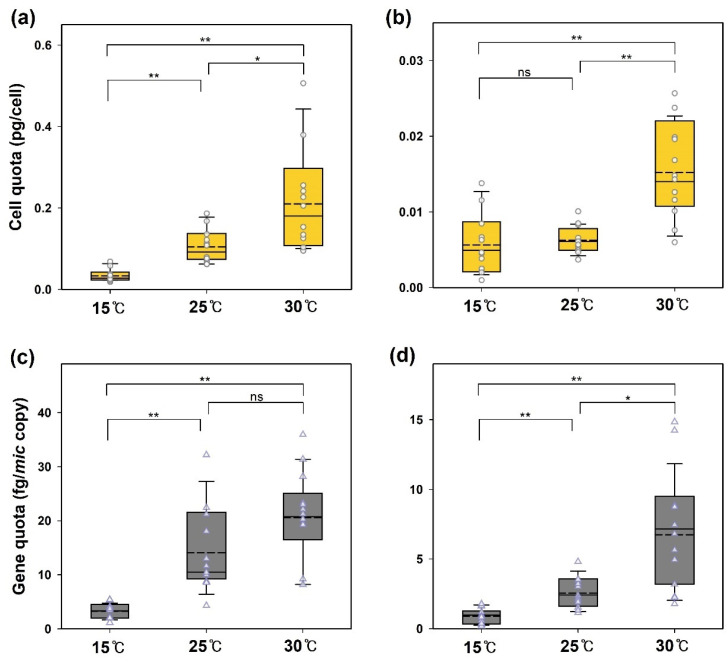
The effect of temperature on the 2-MIB yield of *Pseudanabaena*. 2-MIB cell quotas (pg/cell) in *Pseudanabaena* sp. (**a**) PD34 and (**b**) PD35 and gene quotas (fg/*mic* copy) in (**c**) PD34 and (**d**) PD35 were determined in the exponential phase. In the box plot, the solid line and the dashed line indicate the median and mean value, respectively (ANOVA with the Games–Howell test, *n* = 12. * *p* < 0.05; ** *p* < 0.01; ns = not significant).

Figure 2 shows the effect of temperature on the biosynthesis of 2-MIB by the two strains. The 2-MIB yields per cell and per *mic* DNA copy increased with culture temperature. The average 2-MIB cell quota (pg/cell) for PD34 increased from 0.034 to 0.211 pg/cell as the temperature rose, whereas PD35 showed a significantly lower increase, from 0.006 to 0.015 pg/cell. One-way ANOVA with the Games–Howell test for unequal variance revealed that the cellular 2-MIB yield of PD34 exhibited significant differences (*p* < 0.05 or *p* < 0.01) at each of the three temperatures (Figure 2a). For PD35 cultured at 30 °C, the cell quota was significantly affected (*p* < 0.01) compared to that at the other two temperatures (Figure 2b).

The 2-MIB productivity of cyanobacteria in laboratory cultures at different temperatures has been investigated in various studies. Most studies report that 2-MIB production per microbial unit (MIB mass per Chl-*a* or cell) increases at higher temperatures after the lag phase or during the logarithmic growth period [15,20,21]. In the culture experiments of *Pseudanabaena* sp. FACHB 1277 conducted by Zhang et al. [3] across temperatures ranging from 10 °C to 35 °C, the 2-MIB cell quota followed the described pattern, except at 10 °C, where growth was inadequate. Meanwhile, Wang and Li [20] found a negative correlation between biomass abundance and 2-MIB productivity in temperature-controlled cultures. However, some studies conducted under controlled temperature and light intensity found lower 2-MIB productivity quotas in cultures with growth limitations [15,47]. Despite different results, these studies commonly indicated that high temperatures positively affect the unit yield of 2-MIB.

Although many studies have investigated the 2-MIB productivity of cyanobacterial cells, few have focused on the biosynthesis gene quotas. Wang et al. [48] reported values of 10–45 fg/*mic* copy from *Pseudanabaena* sp. dqh15 cultures, probably grown at similar temperatures. In our experiments, the average 2-MIB gene quota (fg/*mic* copy) ranged from 3.3 to 21.8 fg/copy for PD34 and from 0.9 to 6.8 fg/copy for PD35. These findings suggest a wider range of 2-MIB mass per gene among species and strains. However, both strains exhibited a similar increase (approximately seven-fold) in the gene quota at 30 °C than at 15 °C.

These results demonstrate that temperature consistently influences the production yield of 2-MIB in cyanobacteria, regardless of their growth benefits, individual differences, and even the abundance of the 2-MIB synthase gene. In other words, 2-MIB-producing cyanobacteria in the environment can dominate over a wide range of seasons, but their potential to produce 2-MIB may increase at higher water temperatures. To understand odorant patterns in the field, the synthase gene has been quantified along with cyanobacterial cells as a credible indicator for predicting the source and occurrence of odorants [27,38,49,50]. Previous studies have proposed guidelines for managing odor issues based on the copy number of relevant genes in reservoirs [20,51]. However, field data, including within-site and within-species differences in odorant production per cell and genes, make the determination of the threshold level for alternative values difficult [45]. There may be limitations related to differences in dominant odorant-producing species or the presence of mixed species in freshwater. However, this study still suggests the need for seasonal fractionated management and improving criteria at higher temperatures to predict the occurrence of 2-MIB in the field based on genetic or cellular abundance.

### 3.3. Temperature and Expression Level of the 2-MIB Synthase Gene

RNA gene analysis was conducted to evaluate the effect of temperature stress on 2-MIB biosynthesis in *Pseudanabaena* at the gene level. *mic* gene expression was normalized using 16S rRNA as a control gene to determine the expression level. The calculated RNA expression level provides information about the relative transcriptional regulation of the cell in response to external stress on the target gene.

**Figure 3:**
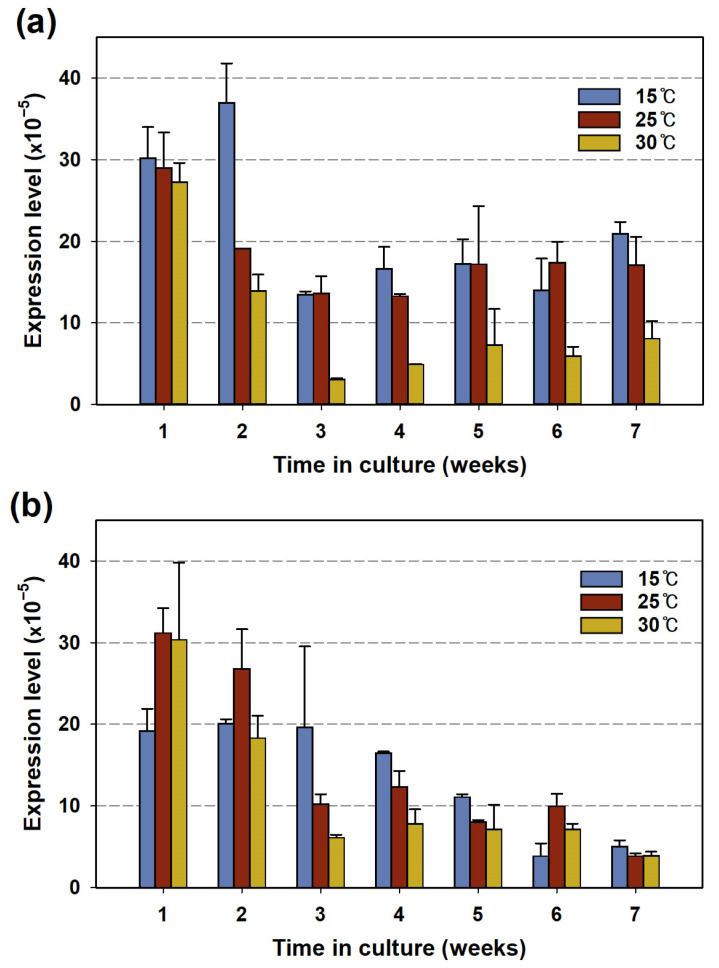
Expression levels of *mic* genes normalized by that of 16S rRNA in *Pseudanabaena* sp. (**a**) PD34 and (**b**) PD35 under the three temperatures.

As shown in Figure 2, the 2-MIB yield per cell and the genomic *mic* gene copy number were proportional to the culture temperature. However, the expression levels of the *mic* gene exhibited an opposing pattern in both strains. In the PD34 culture, the expression level decreased during the first 3 weeks and then gradually increased (Figure 3a). The expression level was higher at low temperatures and was particularly low at 30 °C. The PD35 strain showed a similar trend, except in week 6, where it had low cell density and 2-MIB concentration (Figure 1b), with expression decreasing as the culture temperature increased from week 3 onwards (Figure 3b). In particular, the gene expression level was higher at 15 °C than at the optimal growth temperature (25 °C) for both strains.

A similar observation was made by Shen et al. [21] in a study on 2-MIB-producing *Pseudanabaena foetida* var. *intermedia* NIES-512 based on Chl-*a*. The expression levels showed varying dominance between the 2-MIB Chl-*a* quota and cell growth at different temperatures, with the highest expression level at 15 °C, while population growth and MIB per Chl-*a* were higher at elevated temperatures. Conversely, in another similar study, the expression level of the 2-MIB synthase gene and the biomass of *Pseudanabaena galeata* NIES-512 were observed to increase at higher temperatures [26]. Additionally, few studies have focused on the expression levels of odorant synthase genes normalized to 16S rRNA levels under controlled environmental conditions such as nitrogen and light intensity [11,29,52]. However, no common patterns were found between gene expression, cell growth, and odorant productivity in these studies. Furthermore, relevant studies for a clear interpretation of the external factors, including temperature, on the cellular regulation of 2-MIB synthesis are currently lacking.

Combining our results, the transcription of the odorant synthase gene appears to have an indistinct correlation with metabolic regulation related to cell proliferation. In other words, it is difficult to consider that growth inhibition due to external stress promotes expression of odorant synthase genes. The actual protein productivity in cells, which is a complex result of internal and external factors, is distinct from the presence of rRNA and RNA transcripts that indicate potential synthesis activity [53].

These results suggest that post-transcriptional processes, such as translational regulation and enzyme kinetics, are more crucial factors in 2-MIB synthesis than the transcription step. In the post-transcriptional steps, the formation of 2-MIB was likely limited at low temperatures. For instance, in terpenoid biosynthesis in cyanobacteria under microbial stress, low temperatures reduce enzyme activity, efficiency of protein folding, and membrane fluidity [54,55]. Therefore, future research should focus on understanding the connection between complex metabolic activities for odorant biosynthesis and temperature stress beyond transcriptional regulation.

### 3.4. Relationship between Chl-a and 2-MIB Production

Chl-*a* is a pigment found in certain photosynthetic bacteria and is essential for the growth of cyanobacteria. Cellular Chl-*a* content can vary depending on environmental stress, and differences exist among strains of the same cyanobacteria species [56,57]. The relationship between temperature conditions and Chl-*a* concentration in cells remains unclear and shows different trends among taxa [58,59]. Terpenoids, which are secondary metabolites that require GPP, are closely associated with photosynthetic pigments [60,61]. Chl-*a* has a competitive relationship with 2-MIB or geosmin, sharing a common precursor (GPP or farnesyl diphosphate, FPP) [28]. In our experiment, Chl-*a* was measured to investigate the relationship between Chl-*a* and 2-MIB in *Pseudanabaena* under controlled temperature conditions.

**Figure 4:**
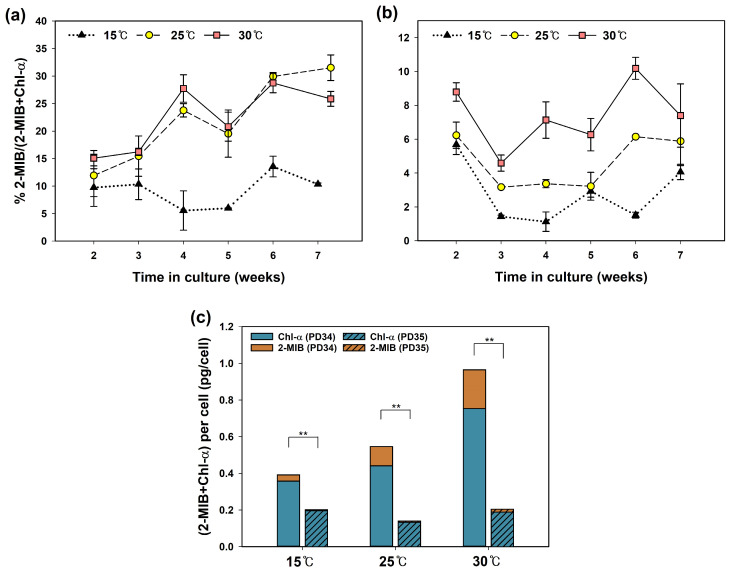
The effect of temperature on the quantitative relationship between 2-MIB and Chl-*a*: proportion of 2-MIB (%) synthesized by *Pseudanabaena* sp. (**a**) PD34 and (**b**) PD35, and (**c**) significant differences between the two strains with regard to the total mass of 2-MIB and Chl-*a* (independent *t*-test; ** *p* < 0.01).

Figure 4 shows the quantitative relationship between Chl-*a* and 2-MIB synthesized by each strain. The 2-MIB proportion (% MIB/(Chl-*a* + MIB)) of PD34 was significantly lower at 15 °C (*p* < 0.01) than at the other two temperatures. For PD35, the 2-MIB% significantly increased (*p* < 0.05) with higher temperatures. These results suggest that high temperatures promote the 2-MIB synthesis pathway in the metabolic competition between Chl-*a* and 2-MIB. Some researchers have shown that excessive light conditions reduce Chl-*a* content [56,62] and instead enhance odorant production [3,63]. Espinosa et al. [63] noted that geosmin, which requires relatively less energy, is synthesized when the need for Chl-*a* decreases depending on light intensity. However, the influence of temperature on the metabolic relationship between the two products has not been studied extensively. In the present experiments, culture temperature affected the 2-MIB synthesis but was not considered to inhibit Chl-*a* synthesis; the amount of cellular Chl-*a* did not decrease at the temperature at which the 2-MIB% increased (Figure 4c).

There was a distinct difference in the proportion of Chl-*a* and 2-MIB between the two strains, with PD35 having less 2-MIB content than PD34. In addition, the total amount of Chl-*a* and 2-MIB produced per cell differed significantly (*p* < 0.01) between the two strains (Figure 4c). Previous studies have reported that the carbon flux in competing pathways with pigments and the limitation of the GPP pool are crucial for determining the production of terpenoids [61,64,65]. Therefore, the difference in 2-MIB proportion % between the two strains can be attributed to the greater allocation of carbon flux to the 2-MIB pathway in PD34 than in PD35. From these results, we identified two characteristics of the cultured *Pseudanabaena* sp. First, the biosynthesis process from the common precursor to 2-MIB was promoted at high temperatures. This supports the fact that high temperatures contribute to the upregulation of 2-MIB through processes other than transcriptional regulation. Second, the difference in 2-MIB production levels between the two strains has been speculated to be due to variations in the GPP pool and resource distribution. These findings suggest that temperature stress significantly impacts the production of 2-MIB in *Pseudanabaena* from genetic and biochemical perspectives. However, further studies are needed to clearly demonstrate the mechanisms by which high temperatures promote 2-MIB synthesis in other cyanobacterial taxa.

## 4. Conclusions

In this study, we show the effect of the culture temperature (15, 25, and 30 °C) on 2-MIB productivity and *mic* gene expression of two strains, *Pseudanabaena* sp. PD34 and PD35. The 2-MIB production yield per cell and *mic* gene (in the DNA sample) in both strains increased across temperatures ranging from 15 °C to 30 °C, regardless of their optimal growth. However, the expression level of the *mic* gene was lowest at 30 °C, suggesting that high temperatures may promote post-transcriptional processes related to the biosynthetic metabolism of 2-MIB. Consistent with this, 2-MIB synthesis was upregulated compared to Chl-*a* at high temperatures through the quantitative relationship of 2-MIB and Chl-*a* in metabolic competition. Additionally, there was a distinct difference in the productivity of 2-MIB between the *Pseudanabaena* strains, although the production patterns and genetic regulation of 2-MIB in the two strains in response to temperature stress were similar. Our findings, which reveal that the characteristics of 2-MIB vary depending on temperature and cyanobacterial strain, provide new insights for managing the complex 2-MIB issues in aquatic resources by utilizing data on the relevant gene and cyanobacteria.

## Data Availability

The data presented in this study are available from the corresponding author upon reasonable request.

## Supplementary Materials

The following supporting information can be downloaded at: https://www.mdpi.com/article/10.3390/cells13161386/s1, Figure S1: Phylogenetic tree based on 16S rRNA gene sequences of *Pseudanabaena* sp. PD34 and PD35 with *Pseudanabaena* strain information obtained from NCBI. The 16S rRNA sequence of *Synechococcus* sp. was used as an outgroup taxon. The tree was constructed using the neighbor-joining method with the maximum composite likelihood model, employing MEGA10 with 1000 bootstrap replications, and bootstrap values (%) are indicated at nodes.
