# Supplementary material for: Effect of Culture Temperature on 2-Methylisoborneol Production and Gene Expression in Two Strains of Pseudanabaena sp
Source: Cells. 2024 Aug 20;13(16):1386. doi: 10.3390/cells13161386 (PMC11352632; doi:10.3390/cells13161386)

Supplementary

Figure S1. Phylogenetic tree based on 16S rRNA gene sequences of *Pseudanabaena* sp. PD34 and PD35 with *Pseudanabaena* strains information obtained from NCBI. The 16S rRNA sequence of *Synechococcus* sp. was used as an outgroup taxon. The tree was constructed using the neighbor-joining method with the maximum composite likelihood model, employing MEGA10 with 1000 bootstrap replications, and bootstrap values (%) are indicated at nodes.

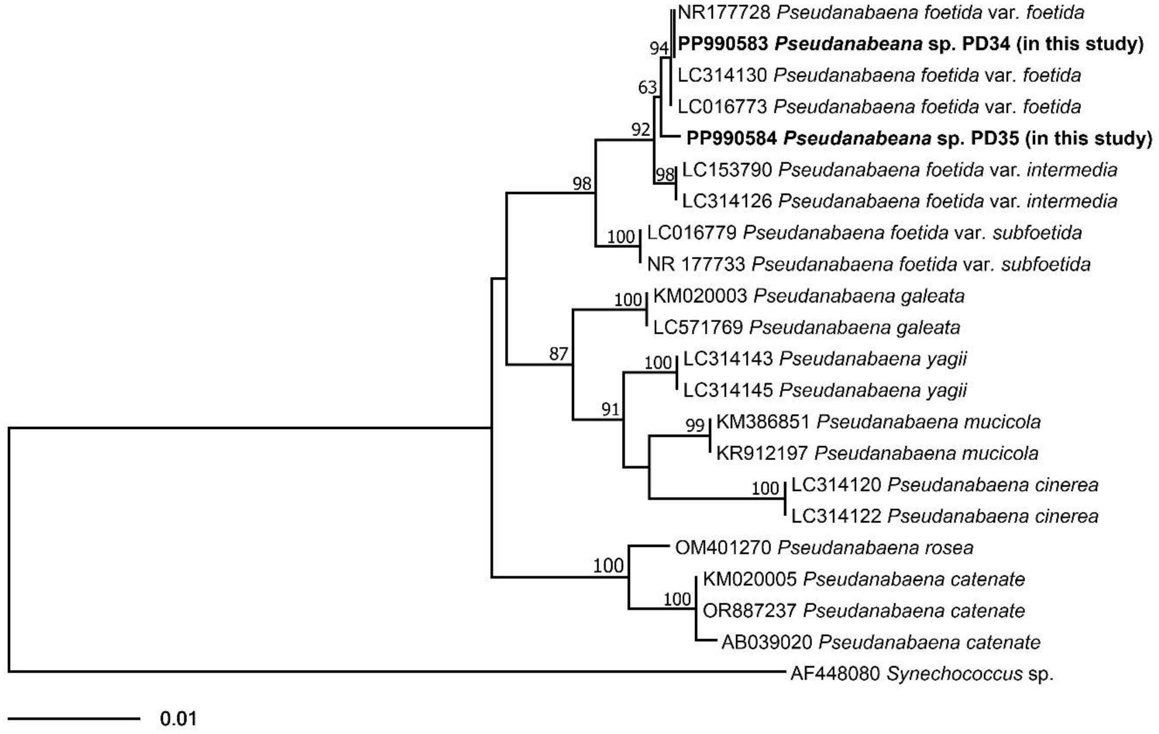

Supplement: Supplementary file 1 [file cells-13-01386-s001.zip › cells-3126919-supplementary.pdf]
